# YOLOv5s-TC: An Improved Intelligent Model for Insulator Fault Detection Based on YOLOv5s

**DOI:** 10.3390/s25164893

**Published:** 2025-08-08

**Authors:** Yingying Yin, Yunpeng Duan, Xin Wang, Shuo Han, Chengyang Zhou

**Affiliations:** College of Information Technology, Jilin Agricultural University, Changchun 130118, China; yyy@jlau.edu.cn (Y.Y.); jarryxin@jlau.edu.cn (X.W.); 19848142825@163.com (C.Z.)

**Keywords:** insulator detection, YOLOv5s, deep learning, computer vision

## Abstract

Insulators play a pivotal role in power grid infrastructure, offering indispensable electrical insulation and mechanical support. Precise and efficient detection of insulator faults is of paramount importance for safeguarding grid reliability and ensuring operational safety. With the rapid advancements in UAV (unmanned aerial vehicle) technology and deep learning, there has been a notable transition from traditional manual inspections to automated UAV-based detection systems. To further enhance detection accuracy, this study conducts a series of systematic improvements to the YOLOv5s model and proposes an advanced intelligent insulator detection model, namely YOLOv5s-TC. Firstly, this new model replaces the C3 (Cross Stage Partial Bottleneck with 3 convolutions) module with Bottleneck Transformers to enhance feature learning ability. Secondly, the CBAM (Convolutional Block Attention Module) is introduced to make the model focus more on the key features of the images, thus improving the target localization ability. Finally, the improved loss function named OSIoU is adopted to further enhance detection accuracy. Comparative experiments demonstrate that YOLOv5s-TC achieves significant performance gains, with mean average precision improvements of 4.4%, 24.5%, and 13.9% over the original YOLOv5s, Faster R-CNN, and SSD models, respectively. The results indicate that YOLOv5s-TC offers superior detection performance and greater reliability for practical power grid inspection applications.

## 1. Introduction

As one of the most complex and critical human-engineered systems, the power grid serves as fundamental energy infrastructure underpinning socioeconomic development. Acting as the significant energy conduit for a nation, it plays a pivotal role in sustaining daily life, industrial operations, and social stability. For power utilities, ensuring secure and reliable grid operation constitutes the paramount operational objective.

**Figure 1:**
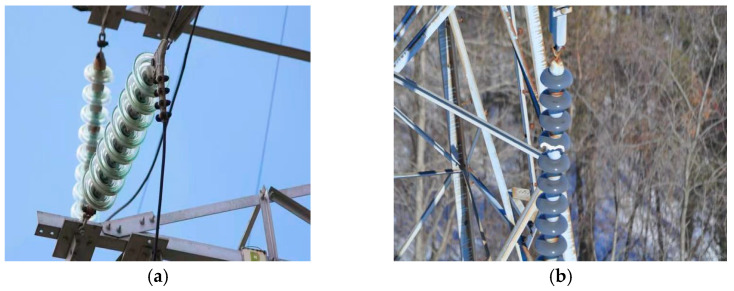
Pictures of insulators. (**a**) Sound insulator. (**b**) Damaged insulator.

Insulators, acting as crucial components in power transmission networks, perform dual core functions: providing mechanical support for conductors and establishing electrical insulation between transmission lines and tower structures. Under actual operating conditions, these components endure prolonged exposure to multiple environmental stressors, including solar radiation, ice accumulation, sandstorms, and high-voltage electrical stresses [1]. Such harsh operational environments accelerate material aging, leading to various failure modes such as string separation, mechanical fractures, and insulation flashover. Figure 1a and Figure 1b present a sound insulator and a damaged insulator, respectively. Compromised insulators significantly impair power transmission quality, with current statistics indicating that approximately 80% of transmission failures originate from insulator defects [2]. Consequently, implementing systematic inspection protocols, preventive maintenance programs, and timely defect remediation through cleaning or replacement emerges as a critical strategy for preventing transmission line failures and ensuring overall grid reliability.

Currently, insulator fault detection is undergoing a significant transformation from traditional manual methods to intelligent UAV-based approaches. The intelligent detection system employs high-definition cameras as primary imaging devices coupled with deep learning algorithms for precise object detection and defect identification. Compared to conventional manual inspection, UAV-based intelligent detection offers substantial advantages by overcoming challenges such as high labor intensity, low efficiency, and significant safety risks. Furthermore, it demonstrates superior adaptability to diverse natural environments, including complex weather conditions and terrains, while reducing power outage frequencies, making it the optimal inspection solution.

To address the challenges of low recognition accuracy and difficult image recognition in intelligent insulator defect detection, this study proposes an enhanced YOLOv5s-TC detection model through improvements to the existing YOLOv5s framework. The proposed model incorporates three key innovations: (1) replacement of the C3 module with Bottleneck Transformers to enhance feature extraction capability, (2) integration of the CBAM to improve feature localization, and (3) implementation of an optimized Scaled Intersection over Union (OSIoU) loss function to boost detection accuracy. Additionally, this research establishes an insulator image dataset that not only expands existing data resources but also yields superior model parameters through training.

## 2. Related Work

In recent years, driven by the escalating and critical need for reliable power grid maintenance, insulator detection technologies have experienced remarkable and revolutionary advancements. Both domestic and international researchers have been devoting substantial and painstaking efforts to the development of intelligent detection methods, effectively harnessing the power of state-of-the-art computer vision and deep learning techniques [3].

On the international front, significant progress has been made in applying deep learning to insulator detection. A novel approach presents a diagnostic procedure using an appropriate PD analyzer with multiple HFCT sensors to carry out efficient insulation condition diagnoses [4]. Kumar et al. focused on developing deep learning algorithms for the detection of cracks in glass insulators [5]. After analyzing large-scale datasets of insulator images, the authors propose and evaluate various deep learning architectures, showing that these methods can achieve high accuracy in crack detection. This study has important implications for improving the reliability of power transmission infrastructure and reducing potential failures caused by insulator defects.

As we mentioned earlier, research on the use of UAV inspection in insulator detection is also quite common. The integration of UAVs with advanced detection algorithms has emerged as a global research hotspot. Liu et al. [6] proposed a novel efficient cross-modality insulator augmentation algorithm for multi-domain insulator defect detection to mimic real complex scenarios. It also alleviates the overfitting problem without adding inference resources. Zhang et al. [7] proposed a lightweight network which introduced the Ghost module into the YOLOv5 backbone and neck to reduce the parameters and model size to enhance the performance of unmanned aerial vehicles. Jeffrey et al. [8] presents an advanced UAV-based multimodal imaging system integrating IR and RGB sensors with deep learning for comprehensive PV defect detection. Souza et al. [9] presented Hybrid-YOLO, an innovative deep learning framework for automated detection and classification of insulator defects in power transmission lines using UAV-captured images. The proposed model integrates a modified YOLO architecture with multi-scale feature fusion, demonstrating enhanced recognition accuracy for various defect types under challenging environmental conditions.

International research teams, such as Patel et al. [10], developed thermal anomaly detection systems for UAV applications. Jae et al. [11] implemented UAV-based thermal imaging for critical nuclear facility components, applying CNN-driven object detection algorithms to pinpoint equipment malfunctions. Johnson et al. [12] proposed a few-shot learning approach for insulator detection in low-resource settings, addressing data scarcity challenges in power line inspections. Experimental results based on UAV-captured images show significant improvements in detection accuracy, offering a practical solution for remote areas with insufficient labeled data.

Recent research has also placed strong emphasis on defect-specific detection methods. Chen et al. [13] proposed an insulator defect detection method called INSU-YOLO based on deep neural networks. Innovative approaches include the self-supervised learning method proposed by Liu et al. [14] and the data augmentation technique for small-sample scenarios developed by Wang et al. [15]. These methods tackle crucial challenges in practical applications.

Wu et al. [16] enhanced FPN feature fusion via cross-layer connections and integrated the ECA module to improve FPN output feature quality, yet accuracy lags behind state-of-the-art methods, requiring further optimization. Qu et al. [17] developed a lightweight directional detector with efficient feature fusion via reduced redundant convolutions, optimized paths, and attention, but accuracy, speed, and training data need improvement. Sun et al. [18] proposed ID-Det (ISNet + IBD), boosting insulator segmentation, but it lacks validation on UAV images under extreme weather (rain, fog, and snow), requiring better robustness and generalization for UAV imagery.

Fang et al. [19] addressed complex backgrounds and small defects in UAV insulator images but performs well only under sufficient illumination, with limited capability in low-light conditions. Ji et al. [20] strengthened multi-scale fusion via ASFF, optimized feature pyramid efficiency with BiFPN_CBAM, and achieved a light weight via ShuffleNetV2, improving accuracy and mean average precision on a self-built dataset, though generalization ability is lacking. Wang et al. [21] modified YOLOv5 to balance performance and speed in high-altitude insulator detection, but small-target accuracy is insufficient. Zhao et al. [22] proposed an improved YOLOv11n model with high accuracy and low complexity, showing that it is suitable for real-time monitoring, yet limited training data hinders generalization. Chai et al. [23] presented FPFS-YOLO, enhancing small-defect feature extraction/fusion via multi-layer networks to improve detection accuracy, but parameter count requires further reduction.

In summary, intelligent insulator detection technology is an indispensable component of smart grids. Existing studies have primarily focused on improving basic algorithms and achieved significant progress. However, several issues remain: some studies use small-scale datasets, which limits the generalization ability of models and makes it impossible to predict their accuracy in large-scale detection; algorithms based on real-time UAV detection are restricted by the computing power and energy consumption of UAVs, leading to insufficiently accurate localization of defect features and thus poor detection precision; and some models have excessive parameters, resulting in slow operation speeds. To address these existing problems, this study is committed to lightweighting the network to reduce computational costs and improve operation speed. In particular, extensive work has been conducted in capturing image features of defective regions, using more effective loss functions, and adding an attention mechanism to the model to obtain a more efficient feature extraction network.

## 3. Improved Model: YOLOv5s-TC

### 3.1. The Architecture of YOLOv5s-TC

The overall structure of YOLOv5s-TC comprises an input layer, a backbone network, a neck network, and an output layer. Compared with YOLOv5s, the Focus module, CSP (Cross Stage Partial) module, and SPP (Spatial Pyramid Pooling) module remain unchanged. The main alterations lie in the number of network layers and parameters.

Based on YOLOv5s, the improvements in YOLOv5s-TC are as follows: a C3TR (C3 Transformer) module and a CCBL (Convolutional attention mechanism Convolutional Block Layer) module are newly introduced, and the loss function is adjusted.

**Figure 2:**
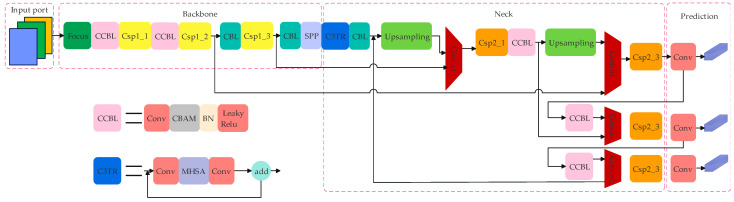
YOLOv5s-TC network architecture.

In the new C3TR module, a Bottleneck Transformers structure is introduced to reduce the computational burden, shorten the model training time, and improve the detection accuracy. The new CCBL module adds a convolutional attention mechanism after the original CBL (Convolutional Block Layer) module, aiming to focus on the key information. The main adjustment to the loss function lies in the redefinition of the distance cost function. Figure 2 shows the network architecture of YOLOv5s-TC.

### 3.2. Replacement of C3 Module with C3TR

This improvement is inspired by BoTs (Bottleneck Transformers). The core of BoT is the BoT block, which is constructed by replacing the spatial 3 × 3 convolution in the ResNet (Residual Network) bottleneck block with an MHSA (Multi-Head Self-Attention) layer.

**Figure 3:**
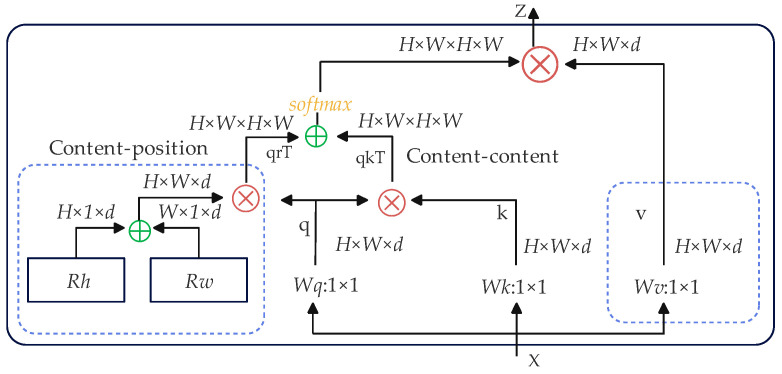
MHSA architecture.

The MHSA module is a key component of the Transformer architecture, playing a significant role in sequential data processing, especially in natural language processing tasks. Its design enables the Transformer to effectively capture long-term dependencies. The MHSA module takes a feature map X of dimensions H×W×d as input, where *H* and *W* are the height and width of the feature map, and *d* is the dimensionality of the feature vector per point. Feature map X generates the query (q), key (k), and value (v) through three 1×1  convolutions. Relative position encodings, *R_h_* and *R_w_*, are introduced to account for the spatial positions of elements. Two attention scores, q^kT^ and q^rT^, are calculated and summed to represent the importance of different positions in the input for output generation. These scores are normalized by the softmax function. The normalized weights are then used to weight-sum and aggregate v, generating the output feature map Z that encapsulates context-aware information for downstream tasks. The MHSA architecture is shown in Figure 3.

In the YOLOv5s-TC model, the 3 × 3 convolutional layer in the ResNet structure is also replaced by the MHSA, and then it is encapsulated into C3TR. Subsequently, the C3TR is further used to replace the C3 module in YOLOv5s. This improvement combines the local perception ability of CNNs with the global perception ability of Transformers, enabling the extraction of useful features from the input images. As a result, the detection accuracy is significantly improved on the premise that the number of model layers, the number of network parameters, the computational load, and the training time have not changed. Therefore, this particular improvement enhances the detection performance of the model without consuming more computing power.

### 3.3. Addition of the Attention Mechanism

The attention mechanism in neural networks is an important technique for enhancing the performance of models in processing sequential data. In 2018, the CBAM attention mechanism model was proposed [24], which aims to improve the performance of convolutional neural networks.

#### 3.3.1. The Design of the Channel Attention Module

**Figure 4:**
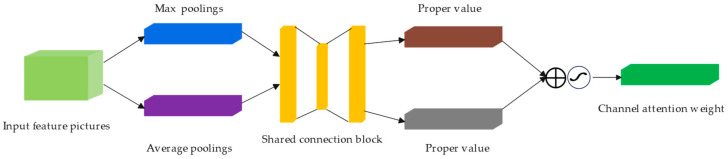
Channel attention module.

The design of the channel attention module in convolutional attention is illustrated in Figure 4.

For each channel, the GAP (Global Average Pooling) calculates the mean value of all spatial positions on that channel, thus compressing the spatial dimension of each channel into a single scalar. Subsequently, a lightweight FC (Fully Connected) network is employed to transform the compressed vector with the purpose of learning the non-linear relationships among channels and generating the channel weights. The FC network consists of two layers. One layer is responsible for dimensionality reduction to decrease the number of parameters and computational cost, while the other layer is tasked with dimensionality increase to restore the number of channels to the original value. The ReLU (Rectified Linear Unit) activation function is utilized between the two layers, and the sigmoid activation function is applied in the last layer to output the weight coefficient of each channel. The values of the weight coefficients all range between 0 and 1. Its expression is shown in Equation (1).

(1) $$Ac(F)=\sigma (MLP(F_{avg}^{c})⊕MLP(F_{max}^{c})),$$

where σ represents the sigmoid activation function, MLP represents the Multi-Layer Perceptron, $F_{avg}^{c}$ represents the feature value obtained after the sample goes through average pooling, $F_{max}^{c}$ represents the feature value obtained after the sample goes through max pooling, and “⊕” represents the addition of features.

Finally, by multiplying the weight of each channel by the features of the corresponding channel in the original feature map, the channels that are more important for the current task are highlighted, while the unimportant channels are suppressed.

#### 3.3.2. The Spatial Attention Module

**Figure 5:**
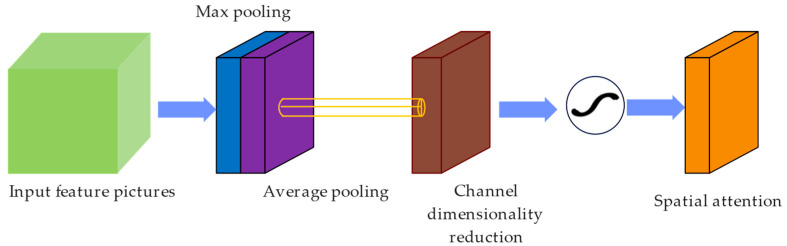
Spatial attention module.

In order to identify the important positions in the feature map, complement the channel attention mechanism, focus on the spatial distribution of the feature map, and suppress the unimportant regions, a spatial attention module is introduced, as shown in Figure 5.

Firstly, we integrate different channel information of the feature map to generate a spatial attention map. This process starts with the compression of the input feature map. Specifically, we use the max- pooling and average pooling operations to aggregate the channel dimension, converting it into a one-dimensional vector feature map of size HW1. Subsequently, we concatenate the results of these two pooling operations, obtaining a two-channel feature map that contains both the maximum and average values. Then, a 7 × 7 convolution operation is applied to reduce the number of channels to 1. Next, a lightweight convolutional network is employed to process the fused feature map. This network is designed to learn spatial dependencies and generate the spatial attention map. For efficiency, it consists of one or more convolutional layers with small-sized kernels such as 3 × 3 or 1 × 1. The final convolutional layer incorporates a sigmoid activation function, which ensures that the values within the attention map range from 0 to 1, effectively representing the significance of each spatial position. Finally, we perform element-wise multiplication between the original feature map and the generated spatial attention map. This operation dynamically adjusts the feature responses at each position, enabling the model to concentrate on crucial spatial regions while mitigating the influence of less important areas. The weighting operation is

(2) $$A_{s}(F)=sigmoid(f_{Conv}^{7*7}([F_{avg}^{S};F_{max}^{S}]))$$

#### 3.3.3. The CBAM Structure Is Integrated into the Backbone Network of YOLOv5s

**Figure 6:**
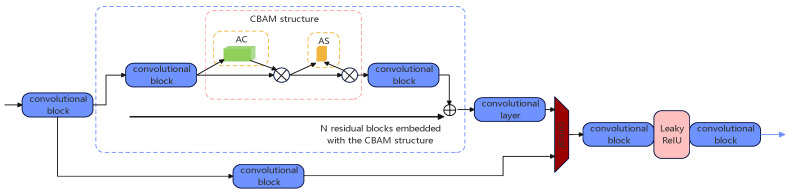
CSP_CBAM_N architecture.

To more effectively enhance the feature extraction of distant targets by the backbone network, we integrate the CBAM structure into the backbone network of YOLOv5s, forming a new backbone feature extraction network, the CSP_CBAM_N structure. This reduces the impact of background noise on images and improves the accuracy of insulator defect detection. The structure diagram is shown in Figure 6.

The specific locations for incorporating the CBAM (Convolutional Block Attention Module) mechanism within the YOLOv5s architecture will be elucidated based on experimental data.

### 3.4. Adjustment and Improvement of the Loss Function

The localization loss function is crucial in object detection. It evaluates the difference between predicted and ground truth bounding boxes, serving as a simple yet effective performance metric. Common localization loss functions include IoU (Intersection Over Union Loss), GIoU (Generalized Intersection Over Union Loss) [25], DIoU (Distance Intersection Over Union Loss) [26], CIoU (Complete Intersection Over Union Loss) [27], and SIoU (Scale Invariant Over Union Loss). Based on their characteristics, we adopted the SIoU function and improved it.

The SIoU loss function contains four elements, namely the angular cost, the distance cost, the shape cost, and the IoU cost. The angular cost is shown in Equation (3), where *α* represents the horizontal angle between the center points of the predicted bounding box and the ground truth bounding box.

(3) $$\Lambda =1-2\times \sin^{2}(arcsin(sin(\alpha )-\frac{\pi }{4}),$$

According to Equation (3), we redefine the distance cost function, as shown in Equation (4).

(4) $$∆=\sum_{t=xy}\left(1-e^{-\gamma \rho_{t}}\right),$$

when *γ* = 2−Λ and *t* = *x*, ρ*_t_* represents the difference between the widths of the ground truth bounding box and the predicted bounding box divided by the square of the width of the minimum enclosing rectangle of the ground truth bounding box and the predicted bounding box. When *t* = *y*, ρ*_t_* represents the difference between the heights of the ground truth bounding box and the predicted bounding box divided by the square of the height of the minimum enclosing rectangle of the ground truth bounding box and the predicted bounding box.

The expressions of the improved shape cost are shown in Equations (5) and (6).

(5) $$\Omega =\;\sum_{t=xy}{\left(1-e^{-\omega_{t}}\right)}^{\theta },$$

(6) $$W\omega \;=\;\frac{\left|\omega -\omega^{gt}\right|}{max(\omega ,\omega^{gt})},\;W_{h}=\;\frac{\left|h-h^{gt}\right|}{max(h,h^{gt})}$$

where (*ω*, *h*) represents the width and height of the predicted bounding box, and ($\omega^{gt}$, $h^{gt}$) represents the width and height of the ground truth bounding box. θ is used to adjust the degree of attention to the shape loss to prevent excessive focus on the shape loss, which may reduce attention to the position of the predicted bounding box. In order to ensure appropriate adjustment, the value range of θ is limited to [2, 6]. The IoU cost function is still used to calculate the degree of overlap between the two bounding boxes.

The improved SIoU loss function is named OSIoU, and its final expression is

(7) $${LOSS}_{ISOoU\;}=1-IoU+\frac{∆+\Omega }{2},$$

In this subsection, the improvements made to YOLOv5s are introduced. In the next subsection, based on the experimental and model training results, the regulatory effect of the loss function and the placement of the attention module will be explored. Additionally, the experimental data of YOLOv5s-TC will be compared with the experimental data of various other mainstream models to determine the superiority of YOLOv5s-TC.

## 4. Experimental Results and Analysis

### 4.1. Dataset Construction

**Figure 7:**
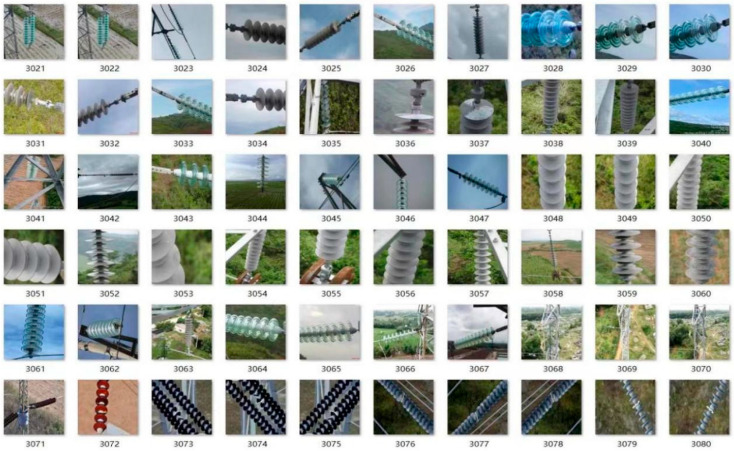
Some samples from the dataset.

The majority of insulator defect images were self-collected using UAVs, while a small portion were sourced from an electric power company. We also expanded the dataset through Mosaic data augmentation methods. The expansion ratio of data augmentation is 4:1. The entire dataset consists of 8700 images, and it is divided into three parts at a ratio of 7:2:1. Specifically, 6090 images are used as the training set, 1740 images as the validation set, and 870 images as the test set. A schematic diagram of some samples in the dataset is shown in Figure 7.

The types of insulator defects in the dataset include four categories: damage, string drop, flashover, and stains. The materials of the insulators cover ceramic insulators, glass insulators, and composite insulators. There are also various suspension methods for the insulators. Additionally, the backgrounds of the dataset include diverse and complex terrains such as mountains, rivers, farmlands, grasslands, and deserts, making it highly representative.

### 4.2. Experimental Parameter Settings

We use Python 3.8 as the programming language, PyTorch1.11.0 as the deep learning framework, NVIDIA GeForce RTX 4060 as the GPU, and we also adopt CUDA 11.4 for acceleration. The hyperparameters used are shown in Table 1. The evaluation indicators include precision and recall rates, mean average precision (mAP), and F1 score (the harmonic mean of the precision and recall rates) [28].

### 4.3. Comparative Experiment of CBAM Location

**Figure 8:**
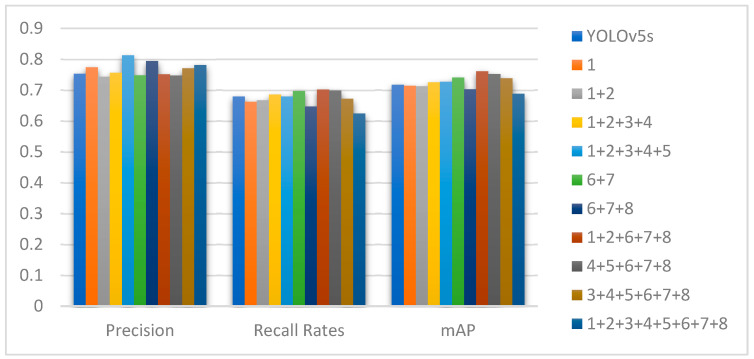
Experimental result of different CBAM adding positions.

In Section 3.3, the specific method for integrating CBAM into YOLOv5s is discussed. The specific insertion positions of CBAM are determined based on experimental data. As shown in Figure 8, where numbers 1–8 correspond to different CBL modules, a comparative analysis of the experimental results reveals that the optimal detection performance is achieved when the CBAM (Convolutional Block Attention Module) is simultaneously added after the convolutional layers of the first, second, sixth, seventh, and eighth CBL modules in YOLOv5s. The mAP value reaches 76.1%, representing a 4.4% improvement over the original YOLOv5s algorithm. This improvement enables the front-placed attention mechanism to not only focus on all key information in insulator images but also, through subsequent attention mechanisms, further emphasize critical information in feature maps, thereby enhancing the detection accuracy of defects in insulator images.

### 4.4. Comparative Experiment of Different Loss Functions

We carried out recognition experiments on the CIoU, DIoU, GIoU, and OSIoU (Optimize SIoU loss function) by employing the YOLOv5s model on our self-constructed dataset. The experimental results are presented in Table 2. Notably, the mAP of the recognition process with the adoption of the OSIoU loss function was 74.4%. Both its precision and mAP surpass those of the other loss functions. Thus, it can be concluded that the modification of the loss function in YOLOv5s yielded certain positive outcomes. The improvements in YOLOv5s make the regression and positioning of defect bounding boxes more accurate, and the model becomes more robust to noise.

### 4.5. Ablation Experiment

Further ablation experiments were conducted after replacing partial C3 modules in the backbone of YOLOv5s with Bottleneck Transformers, introducing the CBAM attention mechanism into the YOLOv5s model, and replacing the loss function with OSIoU. These experiments aimed to analyze the contribution of each individual improvement or pairwise combination among the three modifications to model performance, quantifying the independent effects and synergistic gains of each innovation. The results are presented in Table 3, where A denotes the replacement of partial C3 modules in the backbone with Bottleneck Transformers, B represents the introduced CBAM attention mechanism, and C indicates the adoption of OSIoU as the loss function. As shown in the results, simultaneous improvements in these three aspects lead to increases in precision, recall, and mean average precision, demonstrating that the three modifications exert a synergistic effect on model performance.

The experimental results indicate that each of the three improvements to YOLOv5s contributes marginally independently. The detection performance of pairwise combinations is comparable to that of using only Bottleneck Transformers. However, when all three improvements act synergistically, all evaluation metrics show significant enhancements, demonstrating that their combined effect yields the strongest model gain. Thus, progress has been made in improving YOLOv5s. Subsequent work will compare the detection results of YOLOv5s-TC with other models to verify its superiority.

### 4.6. Comparative Experiment of YOLOv5s-TC

**Figure 9:**
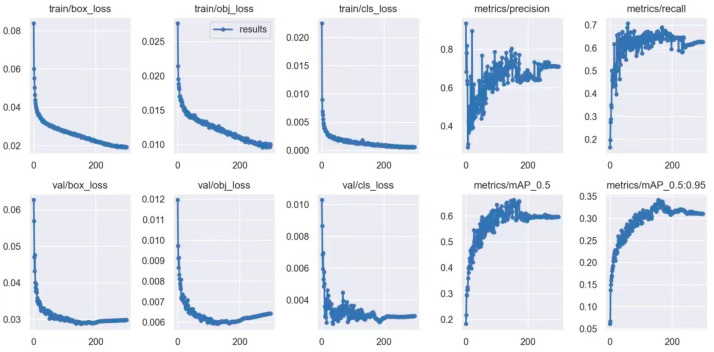
The result of the YOLOv5s-TC model’s training process.

Based on the aforementioned experimental results, the optimization scheme for the loss function and the insertion positions of the CBAM were determined. With this, the construction of the YOLOv5s-TC model was completed. We trained the model using the dataset described in Section 4.1, and the results are shown in Figure 9.

From the training results, it can be seen that various indicators of the YOLOv5s-TC model gradually start to converge after 200 training iterations. When the number of training iterations reaches 300, all the training indicators tend to stabilize. To further verify the superiority of YOLOv5s-TC, we selected YOLOv5s, YOLOv5m, YOLOv5l, and YOLOv5x, along with four current mainstream algorithms, namely Faster R-CNN, SSD, YOLOv3, and YOLOv4, for comparative experiments. The comparison results demonstrate the performance of different insulator defect types and algorithms in terms of precision, recall rate, mAP, and F1 score. Table 4 and Table 5 present the comparative results of various models.

As can be seen from Table 3, the Faster R-CNN algorithm has the lowest average precision compared to several other detection algorithms, and situations such as missed detections and false detections may occur. In contrast, the improved YOLOv5s-TC algorithm exhibits the best detection performance for various types of defects as well as normal conditions. Compared with the original YOLOv5s algorithm, the mean Average Precision (mAP) of the YOLOv5s-TC algorithm increased by 4.1%, 9.4%, 10.8%, and 12% for normal insulators, string-missing insulators, damaged insulators, and flashover insulators, respectively.

Meanwhile, compared with YOLOv5m, which shows the best performance in detecting string-missing insulators among the other seven models, the mAP of the YOLOv5s-TC algorithm increased by 8.5%. When compared with YOLOv5x, which performs best in detecting damaged insulators among the other seven models, the mAP of the YOLOv5s-TC algorithm increased by 10.5%. Moreover, when compared with YOLOv5l, which has the best performance in detecting flashover insulators among the other seven models, the mAP of the YOLOv5s-TC algorithm increased by 10%.

These results indicate that the YOLOv5s-TC model significantly outperforms other algorithms in detecting the three types of insulator defects, namely string drop, damage, and flashover.

Upon examining Table 4, it is evident that the improved algorithm, YOLOv5s-TC, demonstrates superiority over other algorithms with respect to precision, recall rate, and F1 score. This further substantiates that the improved algorithm exhibits more favorable performance in the target detection task, and its detection outcomes are more dependable.

### 4.7. Comparison of Model Structure Loss Values Between YOLOv5s and YOLOv5s-TC

**Figure 10:**
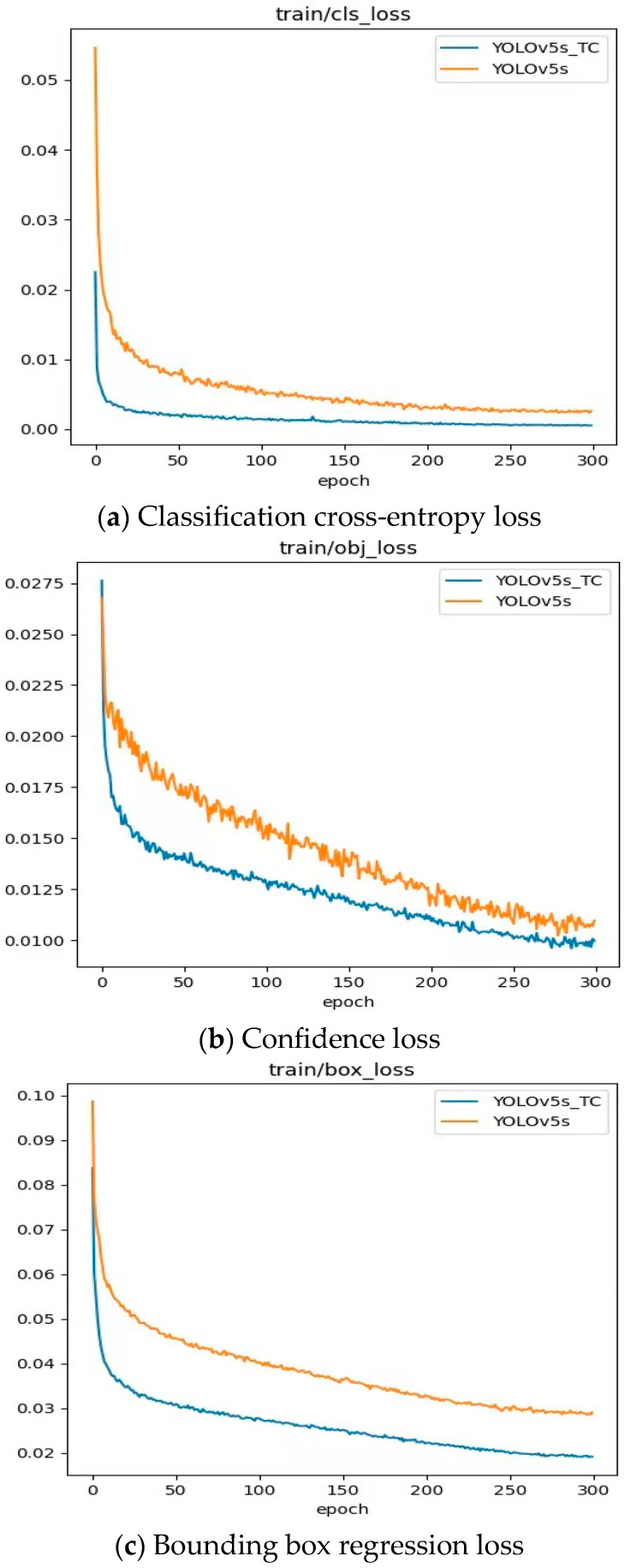
Training loss curve.

We compared the original YOLOv5s model and the improved YOLOv5s-TC model in terms of bounding box regression loss, confidence loss, and classification probability loss, and the results are shown in Figure 10.

It is clearly evident from Figure 10 that in the early stage of training (0 to 10 epochs), the losses of the two models were similar. During the subsequent training process, the three losses of YOLOv5s-TC converged faster, and the loss values were consistently lower than those of the original model. When training reached approximately 250 epochs, the losses of both models converged and stabilized, but the loss value of YOLOv5s-TC was still lower. This indicates that the improved model did not experience overfitting, and it demonstrated reliable performance during both the training and testing stages, once again confirming its reliability and effectiveness in object detection.

### 4.8. Comparison of Computational Efficiency Between YOLOv5s and YOLOv5s-TC

We compared the computational efficiency of the original YOLOv5s and improved YOLOv5s-TC, and the results are presented in Table 6. The data therein show that YOLOv5s-TC has significantly fewer parameters and lower computational costs. Combined with the mAP metrics in Table 5, it is clear that the optimized model reduces computational resources while enhancing the mAP. These findings demonstrate the practical value of the proposed optimization for insulator detection, highlighting the advantages of the improved model.

## 5. Conclusions

This study proposes an improved YOLOv5s-based model for defect detection of insulators in smart grids, with several key innovations: the C3 module in the YOLOv5s backbone is replaced with Bottleneck Transformers, the CBAM attention mechanism is introduced into the YOLOv5s model, and the more effective OSIoU loss function is adopted to replace the original loss function. These improvements significantly enhance detection accuracy and speed while reducing network parameters and computational load.

The experimental results show that the enhanced model outperforms traditional methods, including other earlier YOLOv5 versions and other object detection networks such as Faster R-CNN and SSD. Specifically, the model achieves an average detection precision of 96.5%. Comparative and ablation studies verify the contributions of each module, comprehensively confirming its significant superiority in the field of insulator defect detection. The research findings open up new ideas and lay a solid theoretical foundation for unmanned intelligent inspection of insulators, with high practical engineering application value.

In future research, it is advisable to further explore lightweight designs to adapt to deployment on edge devices. Integrating multimodal data (such as the fusion of infrared and visible light images) can be considered to improve detection capabilities in complex environments. Additionally, introducing distributed learning frameworks like federated learning into the model training process is expected to enable the application of large-scale intelligent power grid inspection while ensuring data privacy.

## Figures and Tables

**Table 1:** Hyperparameter usage statistics.

Serial Number	Hyperparameters	Date
entry 1	epoch	300
entry 2	batch size	100
entry 3	initial learning rate	0.001
entry 4	image size	640 × 640

**Table 2:** Comparison of results with different loss functions.

Network Model	Precision	Recall Rates	mAP	F1
YOLOv5s	0.753	0.679	0.717	0.714
YOLOv5s + CIoU	0.769	0.671	0.727	0.716
YOLOv5s + DIoU	0.740	0.707	0.718	0.723
YOLOv5s + GIoU	0.785	0.690	0.732	0.734
YOLOv5s + OSIoU	0.792	0.798	0.744	0.742

**Table 3:** Ablation experiment results.

Network Model	Precision	Recall Rates	mAP	F1
YOLOv5s	0.753	0.679	0.717	0.714
YOLOv5s + A	0.797	0.705	0.779	0.748
YOLOv5s + B	0.751	0.702	0.761	0.726
YOLOv5s + C	0.792	0.698	0.744	0.742
YOLOv5s + A + B	0.793	0.701	0.768	0.744
YOLOv5s + A + C	0.796	0.707	0.776	0.749
YOLOv5s + B + C	0.794	0.703	0.769	0.746
YOLOv5s + A + B + C	0.818	0.720	0.874	0.766

**Table 4:** Comparison of average detection precision for different types of defects.

Network Model	Good	String Drop	Damage	Flashover
YOLOv5s	0.924	0.802	0.717	0.677
YOLOv5m	0.907	0.811	0.694	0.676
YOLOv5l	0.900	0.794	0.708	0.697
YOLOv5x	0.921	0.773	0.720	0.638
Faster R-CNN	0.825	0.481	0.578	0.584
SDD	0.906	0.636	0.556	0.674
YOLOv3	0.881	0.593	0.651	0.644
YOLOv4	0.897	0.672	0.679	0.658
YOLOv5s-TC	0.965	0.896	0.825	0.797

**Table 5:** Comparison of models’ common indicators in insulator defect detection.

Network Model	Precision	Recall Rates	mAP	F1
YOLOv5s	0.782	0.704	0.730	0.741
YOLOv5m	0.779	0.708	0.720	0.742
YOLOv5l	0.804	0.659	0.715	0.724
YOLOv5x	0.753	0.691	0.710	0.721
Faster R-CNN	0.443	0.636	0.529	0.522
SDD	0.771	0.446	0.635	0.637
YOLOv3	0.741	0.642	0.618	0.688
YOLOv4	0.763	0.661	0.629	0.708
YOLOv5s-TC	0.818	0.720	0.874	0.766

**Table 6:** Comparison of computational efficiency between models.

Network Model	Parameter Quantity	FLOPs/G
YOLOv5s	7,082,435	16.7
YOLOv5m	10,097,773	21.5
YOLOv5l	6,998,467	16.5
YOLOv5x	7,035,652	16.7
Faster R-CNN	18,542,365	42.3
SDD	21,745,631	57.6
YOLOv5s-TC	6,598,029	10.9

## Data Availability

The original contributions presented in this study are included in the article. Further inquiries can be directed to the corresponding author.
